# U-shaped association between neutrophil-percentage-to-albumin ratio and all-cause mortality in adults with hyperlipidemia: A prospective cohort study of NHANES 1999 to 2018

**DOI:** 10.1097/MD.0000000000048365

**Published:** 2026-04-17

**Authors:** Jie Wang, Yanyan Yang, Keting Jiang, Hao Chen, Haibiao Wang

**Affiliations:** a Department of Hepatobiliary and Pancreatic Surgery, Ningbo Medical Center Lihuili Hospital, Ningbo, Zhejiang, China; b Department of Gastrointestinal Surgery, Cixi People’s Hospital, Ningbo, Zhejiang, China.

**Keywords:** cardiovascular disease, hyperlipidemia, mortality, NHANES, NPAR

## Abstract

Hyperlipidemia impacts global mortality, and the neutrophil-percentage-to-albumin ratio (NPAR) is a novel inflammatory marker, but its association with mortality in hyperlipidemic adults is unknown. Using National Health and Nutrition Examination Survey data (1999–2018), this study examined NPAR’s association with all-cause and cardiovascular disease (CVD) mortality in hyperlipidemic adults via multivariate Cox models and restricted cubic splines, with threshold effects and subgroup analyses also evaluated. Among 35,356 participants, NPAR showed a U-shaped link with all-cause mortality (inflection = 11.7; hazard ratio (HR) = 0.94, 95% confidence interval (CI) 0.91–0.98 below; HR = 1.14, 95% CI, 1.12–1.15 above) and a positive linear association with CVD mortality (HR = 1.12, 95% CI, 1.10–1.15). The all-cause mortality risk from higher NPAR was greater in physically inactive individuals. Among US adults with hyperlipidemia, a U-shaped association was identified between NPAR and all-cause mortality, while a linear positive correlation was noted with CVD mortality. An NPAR of 11.7 may be optimal, and physical activity might reduce risks from higher NPAR levels. NPAR may help identify high-risk patients for closer monitoring and targeted prevention strategies.

## 1. Introduction

Hyperlipidemia is a metabolic disorder defined by disproportionately high blood lipid levels, including elevated total cholesterol, triglycerides (TG), and low-density lipoprotein cholesterol (LDL-C), along with declined high-density lipoprotein cholesterol (HDL-C).^[1,2]^ Globally, it has become a widespread concern. Specifically, among US adults, approximately 25% have TG levels ≥ 150 mg/dL, around 17% have HDL-C values under 40 mg/dL, and about 12% have total cholesterol levels over 240 mg/dL.^[3,4]^ With the global obesity, metabolic syndrome, and diabetes epidemics getting worse, the prevalence of hyperlipidemia is just going to get higher.^[5]^ Hyperlipidemia is closely associated with atherosclerotic cardiovascular disease (ASCVD),^[6,7]^ diabetes,^[8]^ and cancer^[9]^; notably, studies have shown that it raises the chance to develop cardiovascular disease (CVD) by 2 to 3 fold.^[10]^ These conditions rank among the world’s major causes of death.^[11]^ Therefore, hyperlipidemia has emerged as a pressing health issue, and it is still imperative to assess hyperlipidemic patients’ prognoses.

Hyperlipidemia often emerges and progresses alongside inflammatory responses.^[12]^ The neutrophil-percentage-to-albumin ratio (NPAR), calculated from neutrophil and albumin levels, is an emerging biomarker of inflammation.^[13]^ Important participants in the inflammatory response, neutrophils, usually show higher amounts under various circumstances.^[14]^ On the other hand, inflammation and oxidative stress frequently have an inverse relationship with albumin levels, and they are often associated with an unfavorable prognosis.^[13,15]^ In patients with CVD, cancer, chronic obstructive pulmonary disease, infections linked to stroke, and hypertension, NPAR has demonstrated strong correlations with predictive outcomes.^[16-21]^ It is yet unknown, though, what role NPAR has as a prognostic factor in hyperlipidemic patients.

Therefore, we hypothesized that NPAR would be associated with all-cause and CVD mortality among adults with hyperlipidemia. Because NPAR is calculated from routinely available laboratory parameters, it could provide a low-cost and widely accessible tool for identifying hyperlipidemic adults at increased risk of adverse outcomes. Clarifying the prognostic value of NPAR in this population addresses an important gap in the current literature and may support earlier risk-stratification, closer follow-up, and targeted prevention strategies in clinical practice.

With the use of data from the National Health and Nutrition Examination Survey (NHANES), this study sought to investigate the relationship between NPAR and mortality among adult US hyperlipidemics, thereby providing important knowledge about disease treatment and preventative tactics.

## 2. Materials and methods

### 2.1. Study population

**Figure 1.**
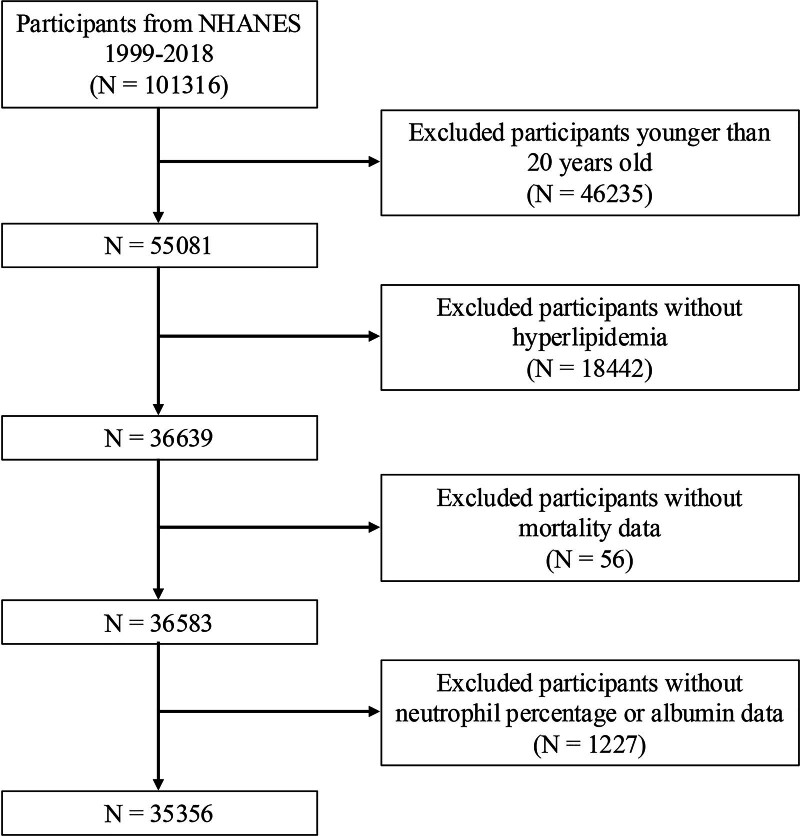
Flow chart of participants selection. NHANES = National Health and Nutrition Examination Survey.

This research analyzed data from NHANES, a comprehensive project conducted by the Centers for Disease Control and Prevention (CDC) to evaluate the health and nutritional status of the US population covering both adults and children.^[22]^ A stratified, multistage, probability-cluster method ensures that NHANES samples reflect the national demographics.^[23]^ Ethical guidelines were strictly followed, with approval from the National Center for Health Statistics Institutional Review Board at the CDC, and all participants provided informed consent.^[24]^ Because the present study is a secondary analysis of publicly available, de-identified NHANES data and involved no collection of new data or use of human tissue, no additional institutional review board approval or additional informed consent was required for this analysis. Our focus was on data from 1999 to 2018, starting with an initial cohort of 1,01,316 individuals. We excluded those under 20 years old (n = 46,235), and further narrowed the field by eliminating subjects without diagnosed hyperlipidemia (n = 18,442). Hyperlipidemia was defined as having TG of 150 mg/dL or more, total cholesterol levels of 200 mg/dL or higher, LDL-C levels of 130 mg/dL or greater, or decreased HDL-C levels (≤40 mg/dL for men and ≤ 50 mg/dL for women), or those on cholesterol-lowering medication.^[25]^ Additional exclusions were made for missing mortality data (n = 56) and incomplete neutrophil percentage or albumin levels (n = 1227). Ultimately, the study encompassed 35,356 participants (Fig. 1).

### 2.2. Assessment of NPAR

We focused on the NPAR as the primary exposure variable, computed by dividing the neutrophil percentage by albumin levels according to the formula^[13]^:


$$\begin{array}{l} \;NPAR=\frac{Neutrophil\;percentage\;\left(\%\right)}{Albumin\;\left(g/dL\right)} \end{array}$$

### 2.3. Ascertainment of mortality

We examined all-cause mortality as the primary endpoint and CVD mortality as the secondary endpoint. Participants’ mortality status was verified using the National Center for Health Statistics’s public-use linked mortality files, with records updated through December 31, 2019. All-cause mortality included deaths due to any reason. For CVD mortality, we identified cases using the International Statistical Classification of Diseases, 10th Revision (ICD-10) codes I00–I09, I11, I13, and I20–I51.^[22]^

### 2.4. Assessment of covariates

We carefully selected covariates to comprehensively evaluate the impact of demographic and health variables on the outcomes. These included gender, age, race, education level, body mass index (BMI), family poverty income ratio (PIR), smoking status, alcohol consumption, physical activity, presence of hypertension and diabetes, antihyperlipidemic treatment, lymphocyte counts, and hemoglobin A1c (HbA1c) levels. Alcohol consumption was specified as the intake of any alcoholic beverage at least once per month over the previous year. Smoking status was defined using the NHANES standard lifetime smoking item; participants were classified as smokers if they reported having smoked ≥ 100 cigarettes in their lifetime, and as nonsmokers otherwise. Consistent with longstanding public health guidance that aerobic activity is typically quantified in bouts lasting at least 10 minutes,^[26]^ physical activity was dichotomized as active or inactive using NHANES questionnaire items that apply this minimum-duration criterion. Participants were classified as active if they reported engaging in moderate or vigorous physical activity for at least 10 minutes during the past 30 days in earlier cycles (1999–2006), or if they reported moderate or vigorous recreational activity for at least 10 minutes in a typical week in later cycles (2007–2018); all others were categorized as inactive. Hypertension, diabetes, and use of antihyperlipidemic medications were determined through self-reported histories via questionnaires.

### 2.5. Statistical analysis

NHANES utilized a complex, multistage, probability sampling design to accurately reflect the noninstitutionalized US civilian population. To support survey-weighted analyses, we employed sample weights, stratification, and clustering techniques. Directly deleting missing values can reduce the sample size, thereby decreasing the statistical power of the study. To mitigate this issue, we employed multiple imputation by chained equations to address the missing values in covariates. We further conducted sensitivity analyses by comparing the results obtained from datasets where missing covariate values were directly deleted with those generated through the multiple imputation method. Baseline characteristics were assessed across quartiles of NPAR, using weighted percentages and means for categorical and continuous variables respectively, each presented with a 95% confidence interval (CI). Categorical variables were examined using survey-weighted Chi-square tests, while continuous variables were examined through survey-weighted linear regression. We explored the association between NPAR and both all-cause and CVD mortality utilizing 3 distinct survey-weighted multivariate Cox proportional hazards models: model 1 unadjusted, model 2 which adjusted for gender, age, race, education level, and family PIR, and model 3 that included a full adjustment for all covariates. We also categorized NPAR into quartiles to explore its linear relationship with mortality using a survey-weighted trend test, and assessed cumulative survival differences across these quartiles through survey-weighted Kaplan–Meier curves and logrank tests. To address potential nonlinear associations, we applied a survey-weighted restricted cubic spline (RCS) regression with Cox proportional hazards models. If RCS regression suggested a nonlinear relationship, we proceeded with threshold effect analysis, initially identifying the inflection point via the trial and error method, followed by a log likelihood ratio test that compared the 1-line Cox model with the 2-piecewise model to evaluate statistical significance. We also conducted subgroup analyses to delve into the relationship between NPAR and mortality across various demographics and lifestyle factors, including gender, age categories, BMI classifications, and statuses of alcohol consumption, smoking, and physical activity. To confirm consistency across these groups, we employed survey-weighted interaction tests. All statistical analyses were performed using R (version 4.3.3, Vienna, Austria) or Empowerstats (version 6.0, Boston), with statistical significance determined by a 2-sided *P*-value of < .05.

## 3. Results

### 3.1. Characteristics of the study participants

Table 1 delineates the baseline characteristics of participants, categorized by quartiles of NPAR. The data is presented as either weighted means or percentages: participants had an average age of 49.58 years, with males constituting 47.74% of the sample, and the overall mean NPAR recorded at 13.89. Analysis revealed that participants in higher NPAR quartiles tended to be older and predominantly female, with elevated BMI and HbA1c levels, coupled with lower family PIR and lymphocyte counts. Additionally, higher NPAR quartiles were associated with increased rates of smoking, hypertension, diabetes, and physical inactivity, alongside a decline in alcohol consumption among these participants.

**Table 1:** Baseline characteristics of participants by neutrophil-percentage-to-albumin ratio.

Characteristics	Neutrophil-percentage-to-albumin ratio				*P*-value
	Q1 (N = 8841)	Q2 (N = 8840)	Q3 (N = 8842)	Q4 (N = 8833)	
Age (yr)	47.31 (46.82, 47.80)	48.54 (48.07, 49.01)	50.59 (50.09, 51.09)	52.07 (51.55, 52.58)	<.001
Gender (%)					<.001
Male	56.19 (54.79, 57.59)	51.37 (50.06, 52.68)	46.43 (45.11, 47.77)	36.03 (34.65, 37.44)	
Female	43.81 (42.41, 45.21)	48.63 (47.32, 49.94)	53.57 (52.23, 54.89)	63.97 (62.56, 65.35)	
Race (%)					<.001
Mexican American	7.95 (6.94, 9.10)	8.87 (7.66, 10.24)	8.17 (7.08, 9.42)	7.63 (6.51, 8.94)	
Other Hispanic	5.84 (4.92, 6.92)	6.35 (5.26, 7.64)	5.24 (4.34, 6.31)	4.96 (4.12, 5.95)	
Non-Hispanic White	63.60 (61.22, 65.92)	70.16 (67.85, 72.37)	73.55 (71.32, 75.67)	72.93 (70.66, 75.08)	
Non-Hispanic Black	14.57 (13.13, 16.13)	7.79 (6.89, 8.79)	6.87 (6.00, 7.85)	8.63 (7.66, 9.71)	
Other Race	8.03 (7.15, 9.01)	6.84 (6.08, 7.69)	6.17 (5.46, 6.96)	5.85 (5.12, 6.68)	
Education level (%)					<.001
<High school	18.35 (17.12, 19.64)	17.34 (16.09, 18.67)	18.20 (17.05, 19.42)	19.29 (18.10, 20.55)	
High school	23.00 (21.55, 24.52)	24.96 (23.60, 26.36)	25.76 (24.50, 27.07)	26.24 (24.97, 27.54)	
>High school	58.66 (56.67, 60.61)	57.70 (56.07, 59.32)	56.04 (54.35, 57.71)	54.47 (52.76, 56.17)	
Family PIR	3.05 (2.99, 3.11)	3.05 (2.99, 3.12)	2.99 (2.92, 3.05)	2.77 (2.70, 2.84)	<.001
BMI (kg/m^2^)	28.31 (28.13, 28.48)	28.97 (28.78, 29.15)	29.85 (29.64, 30.07)	31.57 (31.33, 31.82)	<.001
Alcohol consumption (%)					.001
Yes	61.23 (59.61, 62.83)	58.02 (56.30, 59.72)	54.94 (53.41, 56.45)	48.37 (46.68, 50.06)	
No	38.77 (37.17, 40.39)	41.98 (40.28, 43.70)	45.06 (43.55, 46.59)	51.63 (49.94, 53.32)	
Smoking (%)					<.001
Yes	46.06 (44.40, 47.73)	46.85 (45.42, 48.30)	48.27 (46.87, 49.67)	50.64 (48.87, 52.41)	
No	53.94 (52.27, 55.60)	53.15 (51.70, 54.58)	51.73 (50.33, 53.13)	49.36 (47.59, 51.13)	
Physical activity (%)					<.001
Active	60.38 (58.75, 61.99)	58.52 (56.98, 60.05)	54.81 (53.20, 56.40)	45.57 (43.86, 47.29)	
Inactive	39.62 (38.01, 41.25)	41.48 (39.95, 43.02)	45.19 (43.60, 46.80)	54.43 (52.71, 56.14)	
Hypertension (%)					<.001
Yes	30.80 (29.49, 32.14)	32.56 (31.06, 34.11)	36.20 (34.90, 37.52)	41.64 (40.14, 43.16)	
No	69.20 (67.86, 70.51)	67.44 (65.89, 68.94)	63.80 (62.48, 65.10)	58.36 (56.84, 59.86)	
Diabetes (%)					<.001
Yes	7.08 (6.42, 7.80)	8.69 (7.98, 9.46)	10.54 (9.74, 11.40)	16.45 (15.51, 17.44)	
No	92.92 (92.20, 93.58)	91.31 (90.54, 92.02)	89.46 (88.60, 90.26)	83.55 (82.56, 84.49)	
Antihyperlipidemic treatment (%)					<.001
Yes	20.42 (19.18, 21.72)	20.84 (19.69, 22.04)	24.82 (23.55, 26.14)	27.34 (26.01, 28.71)	
No	79.58 (78.28, 80.82)	79.16 (77.96, 80.31)	75.18 (73.86, 76.45)	72.66 (71.29, 73.99)	
Lymphocyte (10^9^/L)	2.63 (2.58, 2.69)	2.22 (2.20, 2.23)	2.03 (2.00, 2.05)	1.82 (1.80, 1.84)	<.001
HbA1c (%)	5.58 (5.55, 5.61)	5.60 (5.58, 5.63)	5.69 (5.66, 5.71)	5.82 (5.79, 5.85)	<.001

Weighted means (95% CI) for continuous variables, weighted percentages (95% CI) for categorical variables.

BMI = body mass index, CI = confidence interval, HbA1c = hemoglobin A1c, PIR = poverty income ratio, Q = quartile.

### 3.2. Associations between NPAR and mortality

**Figure 2.**
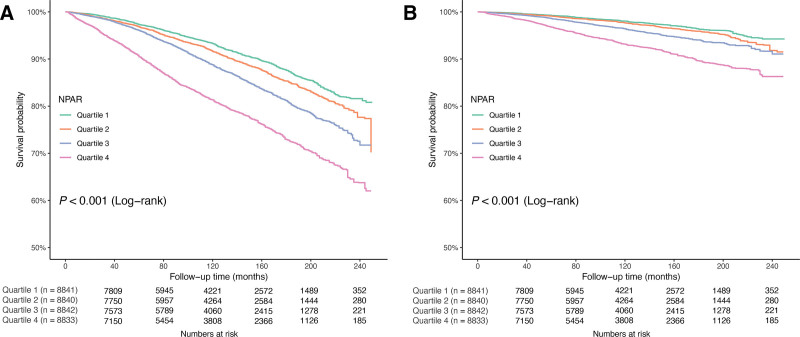
Kaplan–Meier curve for all-cause (A) and CVD (B) mortality by different quartiles of NPAR in adults with hyperlipidemia. *P* values are from logrank tests. CVD = cardiovascular disease, NPAR = neutrophil-percentage-to-albumin ratio.

During the mean follow-up duration of 120.12 months, we documented a total of 5892 deaths, with 1897 of these due to CVD. Table 2 demonstrates a significant positive association between NPAR and both all-cause and CVD mortality across all 3 models of weighted multivariate Cox proportional hazards regression. Specifically, model 3, which included all covariates, showed that each unit increase in NPAR corresponded to an 11% rise in all-cause mortality (HR = 1.11; 95% CI, 1.09–1.12) and a 12% rise in CVD mortality risk (HR = 1.12; 95% CI, 1.10–1.15). Dividing NPAR values into quartiles, survey-weighted trend analyses confirmed that higher NPAR was linked to greater risks of both all-cause and CVD mortality, with *P* for trend < .001 for both outcomes. Sensitivity analyses revealed that the results obtained from datasets with directly deleted missing covariate values also demonstrated a significant positive association between NPAR and both all-cause and CVD mortality (Table S1, Supplemental Digital Content, https://links.lww.com/MD/R727). Figure 2 displays the Kaplan–Meier survival curves, highlighting that the highest quartile (Q4) significantly correlates with increased mortality rates for both all-cause and CVD (*P *< .001 for both).

**Table 2:** Association of neutrophil-percentage-to-albumin ratio with all-cause and cardiovascular disease mortality in adults with hyperlipidemia.

NPAR	Number of deaths	HR (95% CI), *P*-value		
		Model 1	Model 2	Model 3
All-cause mortality				
Continuous	5892	1.13 (1.12, 1.14), <.001	1.12 (1.10, 1.13), <.001	1.11 (1.09, 1.12), <.001
Q1	1044	Reference	Reference	Reference
Q2	1177	1.20 (1.07, 1.34), .002	1.13 (1.01, 1.26), .028	1.13 (1.02, 1.26), .024
Q3	1556	1.57 (1.43, 1.72), <.001	1.27 (1.17, 1.38), <.001	1.26 (1.16, 1.36), <.001
Q4	2115	2.58 (2.35, 2.83), <.001	1.87 (1.73, 2.03), <.001	1.78 (1.64, 1.93), <.001
* P* for trend		<.001	<.001	<.001
CVD mortality				
Continuous	1897	1.15 (1.13, 1.17), <.001	1.14 (1.12, 1.16), <.001	1.12 (1.10, 1.15), <.001
Q1	318	Ref	Ref	Ref
Q2	362	1.23 (1.03, 1.47), .021	1.17 (0.98, 1.38), .074	1.13 (0.95, 1.33), .167
Q3	506	1.69 (1.42, 2.01), <.001	1.35 (1.15, 1.57), <.001	1.26 (1.08, 1.46), .003
Q4	711	3.09 (2.65, 3.61), <.001	2.13 (1.84, 2.47), <.001	1.86 (1.60, 2.17), <.001
* P* for trend		<.001	<.001	<.001

Model 1: no covariates were adjusted. Model 2: age, gender, race, education level, and family PIR were adjusted. Model 3: age, gender, race, education level, family PIR, BMI, alcohol consumption, smoking, physical activity, hypertension, diabetes, antihyperlipidemic treatment, lymphocyte, and HbA1c were adjusted.

BMI = body mass index, CI = confidence interval, CVD = cardiovascular disease, HbA1c = hemoglobin A1c, HR = hazard ratio, NPAR = neutrophil-percentage-to-albumin ratio, PIR = poverty income ratio, Q = quartile.

### 3.3. Detection of nonlinear relationships

**Figure 3.**
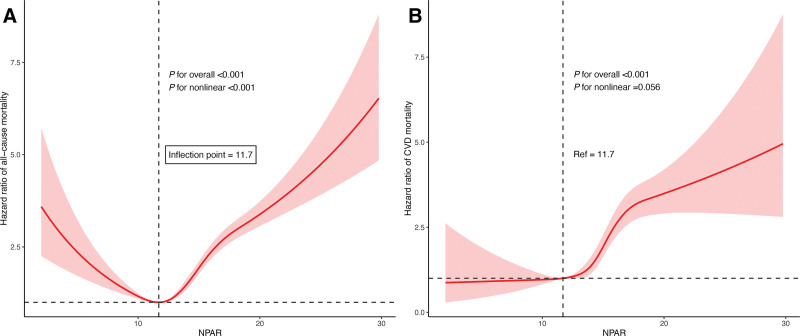
Restricted cubic splines were utilized to evaluate the hypothesis of potential nonlinear relationships between NPAR and all-cause (A) and CVD (B) mortality in adults with hyperlipidemia. Solid lines represent adjusted hazard ratios and shaded areas represent 95% confidence intervals, estimated from Cox proportional hazards models adjusted for age, gender, race, education level, family PIR, BMI, alcohol consumption, smoking, physical activity, hypertension, diabetes, antihyperlipidemic treatment, lymphocyte, and HbA1c. The horizontal dashed line denotes a hazard ratio of 1.0, and the vertical dashed line marks the reference value (NPAR = 11.7). BMI = body mass index, CVD = cardiovascular disease, HbA1c = hemoglobin A1c, NPAR = neutrophil-percentage-to-albumin ratio, PIR = poverty income ratio.

Utilizing an RCS regression with Cox proportional hazards models, our analysis disclosed a significant U-shaped relationship between NPAR and all-cause mortality, as illustrated in Figure 3A. However, no significant nonlinear association was found with CVD mortality (Figure 3B). To further investigate, we performed a threshold effect analysis on the impact of NPAR on all-cause mortality. The log likelihood ratio test showed significant results (*P* < .001), suggesting that a 2-piecewise Cox proportional hazards model provided a better fit for describing the relationship between NPAR and all-cause mortality, detailed in Table 3. We identified 11.7 as the inflection point for NPAR. Below this threshold, an increase in NPAR was significantly linked with a decreased risk of all-cause mortality (HR = 0.94; 95% CI, 0.91–0.98). Conversely, when NPAR exceeded 11.7, higher NPAR values were significantly linked to greater risk of all-cause mortality (HR = 1.14; 95% CI, 1.12–1.15). Consistent findings were observed in sensitivity analyses, which also identified 11.7 as the inflection point for the NPAR–all-cause mortality association (Table S2, Supplemental Digital Content, https://links.lww.com/MD/R727).

**Table 3:** Threshold effect analysis of neutrophil-percentage-to-albumin ratio on all-cause mortality in adults with hyperlipidemia.

	HR (95% CI), *P*-value
NPAR	1.11 (1.10, 1.12), <.001
Inflection point	
<11.7	0.94 (0.91, 0.98), .002
>11.7	1.14 (1.12, 1.15), <.001
*P* for log likelihood ratio test	<.001

The model was adjusted for age, gender, race, education level, family PIR, BMI, alcohol consumption, smoking, physical activity, hypertension, diabetes, antihyperlipidemic treatment, lymphocyte, and HbA1c.

BMI = body mass index, CI = confidence interval, HbA1c = hemoglobin A1c, HR = hazard ratio, NPAR = neutrophil-percentage-to-albumin ratio, PIR = poverty income ratio.

### 3.4. Subgroup analyses

**Figure 4.**
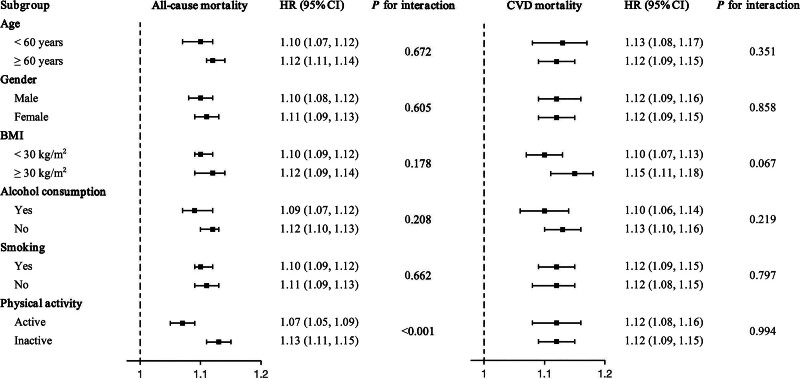
Subgroup analysis of the associations between NPAR and all-cause and CVD mortality in adults with hyperlipidemia. HRs were estimated using multivariable Cox proportional hazards models adjusted for age, gender, race, education level, family PIR, BMI, alcohol consumption, smoking, physical activity, hypertension, diabetes, antihyperlipidemic treatment, lymphocyte, and HbA1c. Squares indicate HR point estimates and horizontal lines represent 95% CIs; the vertical dashed line denotes HR = 1.0. *P* for interaction values were derived from interaction tests to evaluate effect modification across subgroups. BMI = body mass index, CI = confidence interval, CVD = cardiovascular disease, HbA1c = hemoglobin A1c, HR = hazard ratio, NPAR = neutrophil-percentage-to-albumin ratio, PIR = poverty income ratio.

Figure 4 illustrates the outcomes of subgroup analyses and survey-weighted interaction tests aimed at exploring the association between NPAR and all-cause and CVD mortality across diverse demographic subsets. Only physical activity showed a significant interaction for all-cause mortality (*P* for interaction < .001); no other subgroup interactions were significant. Specifically, among physically active participants, a unit increase in NPAR corresponded to a 7% increase in the risk of all-cause mortality (HR = 1.07; 95% CI, 1.05–1.09). Physically inactive participants experienced a 13% increase in all-cause mortality risk for each NPAR unit increase (HR = 1.13; 95% CI, 1.11–1.15). Further subgroup analyses by age, gender, BMI, alcohol use, and smoking status confirmed that the relationships between NPAR and both all-cause and CVD mortality were stable across these factors.

## 4. Discussion

Previous research has demonstrated the predictive significance of NPAR, a novel inflammatory biomarker that is easily obtained and cost-effective across a range of chronic conditions. Elevated NPAR levels were shown by Liu et al to be substantially linked to increased all-cause and CVD mortality in persons with hypertension.^[13]^ Yu et al found that NPAR exhibited superior predictive value for mortality compared to low-density lipoprotein cholesterol-to-high-density lipoprotein cholesterol ratio, neutrophil-to-lymphocyte ratio, monocyte-to-lymphocyte ratio, and platelet-to-lymphocyte ratio.^[27]^ In severely sick patients, NPAR was also found to be an independent predictor of death. According to research by Lin et al, patients with acute myocardial infarction who had a high NPAR level upon ICU admission had an independent relationship with 180- and 365-day all-cause mortality, with predictive performance comparable to the sequential organ failure assessment score.^[28]^ In critically ill patients in coronary care units, Cai et al found a nonlinear relationship between NPAR and in-hospital mortality; that is, when NPAR fell below 1.65, there was a negative correlation with mortality; however, as NPAR exceeded 1.65, the risk of in-hospital mortality rose with increasing NPAR levels.^[29]^ The findings of our investigation are somewhat supported by these studies.

Hyperlipidemia leads to lipid accumulation in arterial walls, initiating atherosclerosis – a process closely linked to inflammatory responses. This inflammation attracts immune cells like neutrophils to plaque sites, with elevated neutrophil levels reflecting this heightened state.^[30]^ Plaque instability is exacerbated by inflammation, increasing the likelihood of plaque rupture and the risk of acute cardiovascular events including heart attacks and strokes.^[31]^ Neutrophils release preformed granule proteins like myeloperoxidase and matrix metalloproteinases, which contribute to myocardial injury.^[32]^ Additionally, interactions between neutrophils and platelets promote atherothrombosis.^[33]^ Albumin, the most prevalent protein in circulation, binds and transports a variety of medications and chemicals, maintaining circulatory system function.^[34]^ It reduces platelet aggregation through mechanisms related to its antioxidant effects in a dose-dependent manner.^[35]^ Hypoalbuminemia indicates malnutrition and the severity of underlying diseases such as infections, cancer cachexia, and liver dysfunction.^[36-38]^ Chronic systemic inflammation can impair immune function, increasing susceptibility to infections and sepsis, which can be fatal. Moreover, inflammation contributes to the initiation and progression of certain cancers by promoting DNA damage, cellular proliferation, and inhibiting apoptosis.^[39]^

Our study uncovered a U-shaped correlation between NPAR and all-cause mortality in adults with hyperlipidemia, indicating that decreased NPAR below the inflection point of 11.7 is associated with increased mortality. The mechanisms behind this association are not fully understood. One possible explanation is that individuals with comorbidities that increase the risk of death, such as agranulocytosis, have very low NPAR values, which negatively impact prognosis.^[29]^ Neutropenia is a known risk factor for bacterial infections, leading to severe sepsis and septic shock.^[40]^ A maladaptive low NPAR response to physiological stress might also result in unfavorable outcomes.^[41]^ Additionally, it has been shown that elevated blood albumin levels are linked to a higher prevalence of diseases including diabetes and chronic renal disease, which contribute to atherosclerosis development and higher mortality risk.^[34,42]^ Dehydration is the primary cause of higher blood albumin levels > 4.5 mg/dL, despite the fact that high-protein meals can marginally boost albumin production.^[36]^ Dehydration can worsen illness progression or recovery from surgery and is linked to increased bradyarrhythmias and frailty.^[43,44]^ A meta-analysis by Edmonds and colleagues showed the 30-day mortality rate is over twice as high in older adults who are dehydrated upon hospital admission compared to those who are euhydrated.^[45]^

The subgroup analyses indicated that physical activity moderates the positive link between NPAR and all-cause mortality in hyperlipidemic adults, suggesting that physical activity may provide a protective effect against the risks associated with elevated NPAR levels. The protective impact of physical activity is linked to its anti-inflammatory properties, primarily through 3 mechanisms: increasing the output and release of anti-inflammatory cytokines from active skeletal muscles; lowering visceral fat mass; and reducing Toll-like receptor expression on monocytes and macrophages, which inhibits downstream inflammatory responses.^[46-48]^. Additionally, physical activity helps mitigate comorbidities by maintaining a healthy weight, reducing obesity-related complications, improving insulin sensitivity, aiding in blood glucose management, and reducing the risk of diabetes-related complications.^[49,50]^

This study has several strengths. First, it pioneers the investigation into the nonlinear correlation between NPAR and mortality in adults with hyperlipidemia. Second, utilizing a large and nationally representative US sample allows for the generalization of the results to a wider population. Third, we enhanced the reliability of our findings through regression analyses that adjusted for various confounding covariates. Additionally, to verify the consistency of the association across various demographics, subgroup analyses were conducted. However, potential limitations should be noted. The main limitation stems from the study’s observational design. Reverse causality cannot be excluded, as preexisting or subclinical disease may influence baseline neutrophil percentage and albumin levels, thereby affecting NPAR and its observed association with mortality. Furthermore, while we accounted for multiple covariates to mitigate potential confounders, it is possible that not all confounding factors were completely eliminated. Smoking status was based on a self-reported lifetime threshold (≥100 cigarettes), which may not fully capture light or intermittent smoking and could lead to some exposure misclassification. Physical activity was self-reported and dichotomized using broad NHANES questions; this may not fully capture activity dose and could result in misclassification. Several variables were self-reported in NHANES, which may be subject to recall or reporting errors and could result in misclassification. Therefore, these findings should be considered exploratory and are not yet suitable for clinical implementation or risk-stratification in routine practice. Additional research is essential to verify these findings and elucidate the underlying biological mechanisms.

## 5. Conclusions

This study, utilizing a nationally representative US sample, identified a U-shaped correlation between NPAR and all-cause mortality, suggesting that an NPAR of 11.7 may be considered safe for adults with hyperlipidemia. Physical activity may offer protective effects against risks associated with elevated NPAR levels. Higher NPAR levels correspond with increased CVD mortality risk without exhibiting a nonlinear relationship. These findings indicate that NPAR is a valuable predictor of all-cause and CVD mortality in hyperlipidemic adults.

## Acknowledgments

We thank all participants in our study.

## Author contributions

**Conceptualization:** Jie Wang, Keting Jiang.

**Formal analysis:** Hao Chen

**Investigation:** Hao Chen.

**Methodology:** Jie Wang

**Supervision:** Haibiao Wang.

**Writing – original draft:** Jie Wang.

**Writing – review & editing:** Yanyan Yang, Keting Jiang, Hao Chen, Haibiao Wang.

## Supplementary Material


