# Supplementary material for: U-shaped association between neutrophil-percentage-to-albumin ratio and all-cause mortality in adults with hyperlipidemia: A prospective cohort study of NHANES 1999 to 2018
Source: Medicine (Baltimore). 2026 Apr 17;105(16):e48365. doi: 10.1097/MD.0000000000048365 (PMC13095325; doi:10.1097/MD.0000000000048365)
Supplement: Supplementary file 1 [file medi-105-e48365-s001.pdf]

Supplementary table 1 Association of neutrophil-percentage-to-albumin ratio with all-cause and cardiovascular disease mortality in adults with hyperlipidemia (excluding cases with missing covariate, N=21103)

NPAR	Number of deaths	HR (95% CI), *P* value		
		Model 1	Model 2	Model 3
All-cause mortality				
Continuous	3431	1.13 (1.12, 1.15), <0.001	1.11 (1.10, 1.13), <0.001	1.10 (1.08, 1.12), <0.001
Q1	600	Ref	Ref	Ref
Q2	710	1.30 (1.12, 1.51), 0.001	1.23 (1.07, 1.41), 0.003	1.23 (1.11, 1.36), <0.001
Q3	895	1.57 (1.38, 1.79), <0.001	1.31 (1.16, 1.47), <0.001	1.27 (1.17, 1.37), <0.001
Q4	1226	2.62 (2.31, 2.96), <0.001	1.92 (1.71, 2.16), <0.001	1.79 (1.66, 1.94), <0.001
P for trend		<0.001	<0.001	<0.001
CVD mortality				
Continuous	1121	1.17 (1.14, 1.20), <0.001	1.16 (1.13, 1.19), <0.001	1.13 (1.10, 1.16), <0.001
Q1	180	Ref	Ref	Ref
Q2	220	1.42 (1.12, 1.78), 0.003	1.34 (1.08, 1.67), 0.009	1.28 (1.03, 1.60), 0.026
Q3	297	1.84 (1.45, 2.34), <0.001	1.51 (1.21, 1.89), <0.001	1.37 (1.10, 1.72), 0.005
Q4	424	3.38 (2.75, 4.16), <0.001	2.40 (1.98, 2.90), <0.001	2.03 (1.65, 2.49), <0.001
P for trend		<0.001	<0.001	<0.001

Model 1: no covariates were adjusted. Model 2: age, gender, race, education level, and family PIR were adjusted. Model 3: age, gender, race, education level, family PIR, BMI, alcohol consumption, smoking, physical activity, hypertension, diabetes, antihyperlipidemic treatment, lymphocyte, and HbA1c were adjusted. Abbreviation: NPAR, neutrophil-percentage-to-albumin ratio; CVD, cardiovascular disease; HR, hazard ratio; CI, confidence interval; Q, quartile; PIR, poverty income ratio; BMI, body mass index; HbA1c, hemoglobin A1c.

Supplementary table 2 Threshold effect analysis of neutrophil-percentage-to-albumin ratio on all-cause mortality in adults with hyperlipidemia (excluding cases with missing covariate, N=21103)

	HR (95% CI), *P* value
NPAR	1.10 (1.08, 1.12), <0.001
Inflection point	
<11.7	0.95 (0.90, 0.99), 0.017
>11.7	1.13 (1.12, 1.15), <0.001
P for log likelihood ratio test	<0.001

The model was adjusted for age, gender, race, education level, family PIR, BMI, alcohol consumption, smoking, physical activity, hypertension, diabetes, antihyperlipidemic treatment, lymphocyte, and HbA1c. Abbreviation: NPAR, neutrophil-percentage-to-albumin ratio; HR, hazard ratio; PIR, poverty income ratio; BMI, body mass index; HbA1c, hemoglobin A1c.
